# Detection of colistin resistant Klebsiella pneumonia co-producing extended spectrum, AmpC beta lactamase and carbapenemase in a tertiary hospital in Nigeria

**DOI:** 10.1186/2047-2994-4-S1-P129

**Published:** 2015-06-16

**Authors:** I Yusuf, SM Qabli, AM Magashi, AI Balarabe, A Kabir, MR Kabir, ES Olivia, R Abbas

**Affiliations:** 1 Microbiology Unit, King Abdulaziz University, Jeddah, Saudi Arabia; 2 Microbiology, Bayero University Kano, Nigeria, Kano, Nigeria; 3 Microbiology, Delta State University, Abraka, Nigeria; 4 Microbiology, Bayero University, Kano, Kano, Nigeria

## Introduction

From 2011 to date, there are increasing detections of carbapenem resistant *Klebsiella pneumoniae* (CRKP) in a 700 bed capacity general hospital in Kano, Nigeria. CRPK infections are very difficult to treat. Colistin is one of reserved antibiotics for treating CRPKs. Of recent, invitro colistin resistant strains of CRKP were detected by disc diffusion method using 10 *μ*g colistin discs (Oxoid, UK) according to the CLSI guidelines.

## Objectives

To test the susceptibility of CRKP invitro to colistin and screen the colistin resistant isolates for extended spectrum beta-lactamase (ESBL), AmpC and Metallo beta lactamase (MBL) production.

## Methods

Susceptibility of 34 CRPK to colistin was determined using disc diffusion method. Colistin resistant strains were concurrently screened phenotypically for ESBL and MBL according to CLS1 2012 breakpoints using double disk synergy test and modified Hodge test respectively. AmpC was detected using AmpC disk test.

## Results

Result shows that 6 out of 34 CRPKs (17.6%) were resistant to colistin (Interpretative criteria: resistant ≤ 11mm). Five CRPK produced ESBL and AmpC, and 3 produced MBL type of carbapenemase. Co-production of ESBL and AmpC was detected in 4 of the isolates, ESBL and MBL in 3, AmpC and MBL in 2. ESBL, AmpC and MBLs were detected concurrently in 3 CRPK. Five out of the 6 CRKP (83.3%) were isolated from urine and cathertips, while the remaining one was from wound.

## Conclusion

The study indicates that, colistin resistant CRPK strains have emerged in the hospital. Co-production of two or three of the beta lactamase enzymes by many of the isolates is worrisome, since it further narrow down treatment options.

## Disclosure of interest

None declared.
